# Severe Bronchospasm-Induced Myocardial Injury Unmasking a Previously Undiagnosed Myocardial Bridge: A Case Report

**DOI:** 10.7759/cureus.104737

**Published:** 2026-03-05

**Authors:** Brandon S Bharat, Jenny Joseph, Karen Halasan

**Affiliations:** 1 Department of Medicine, Norwalk Hospital, Norwalk, USA; 2 Department of Pulmonary and Critical Care Medicine, Norwalk Hospital, Norwalk, USA

**Keywords:** antibiotics, bronchospasm, cardiology, congenital, critical care, iatrogenic, myocardial bridge, nstemi, pulmonary critical care

## Abstract

Severe bronchospasm resulting in acute respiratory failure may precipitate cardiovascular complications due to myocardial oxygen supply-demand mismatch. A myocardial bridge is a typically benign congenital coronary anomaly that may become clinically significant during periods of tachycardia and increased contractility. We report a case of acute hypoxic hypercapnic respiratory failure due to severe bronchospasm temporally associated with ceftriaxone administration, complicated by myocardial injury attributed to an incidentally identified myocardial bridge. This case highlights the diagnostic complexity of acute dyspnea and underscores the importance of recognizing myocardial bridge during physiologic stress, a phenomenon that has rarely been reported in prior case reports.

## Introduction

Acute respiratory failure requiring endotracheal intubation has a broad differential, including infection, obstructive lung disease, environmental exposures, medication-induced reactions, and bronchospasm [1-3]. The etiologies of bronchospasm are often multifactorial. Cephalosporins are widely used for suspected community-acquired infections and are generally well tolerated; however, hypersensitivity reactions, such as bronchospasm and anaphylaxis, have been reported, particularly in patients with prior beta-lactam exposure [1-3]. Severe bronchospasm can often precipitate cardiovascular stress due to tachycardia and subsequent hypoxemia.

A myocardial bridge is a benign coronary malformation wherein segments of the artery are covered by segments of myocardium. Bridging is typically asymptomatic and often goes undetected, although under periods of hemodynamic instability can produce myocardial damage, which can manifest as angina and masquerade as a myocardial injury [4,5].

We present a rare case of severe bronchospasm leading to acute respiratory failure complicated by myocardial injury in the setting of an incidentally found myocardial bridge, an association not previously described in the literature.

## Case presentation

**Figure 1:**
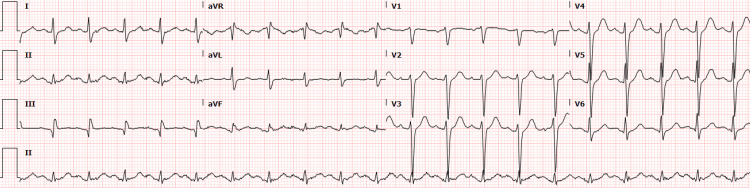
Initial electrocardiogram without any evidence of dynamic ST-segment changes.

A 59-year-old Caucasian male with a past medical history of obstructive sleep apnea (intolerant to continuous positive airway pressure (CPAP)), former smoker, and active vape use presented in the setting of worsened dyspnea, wheeze, and cough. Prior to presentation, he completed a course of amoxicillin-clavulanate with minimal improvement in his symptoms. At the time of admission, he denied a diagnosis of asthma but reported a possible mild antibiotic allergy in early adolescence. He reported worsening of his symptoms throughout the day before developing an intermittently productive cough. He was acutely treated with ceftriaxone, azithromycin, albuterol, and DuoNebs at 2309. On arrival, he was tachycardic without evidence of respiratory distress. Initial labs were significant for leukocytosis of 14.3 × 109/L, a D-dimer of 520 ng/mL, and a troponin of 10 ng/L. An initial electrocardiogram (EKG) demonstrated sinus tachycardia without dynamic ST-segment changes (Figure 1).

**Figure 2:**
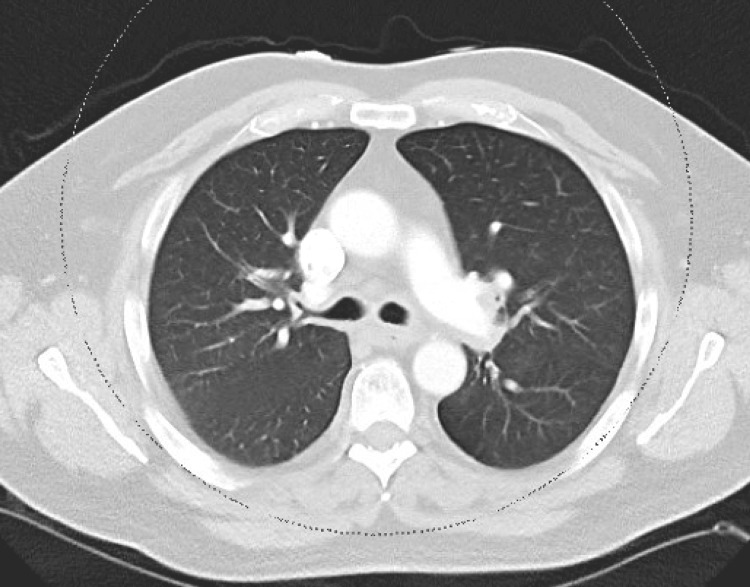
Computed tomography angiography of the chest without evidence of pulmonary embolism.

A computed tomography (CT) angiogram of the chest showed no evidence of pulmonary embolism (Figure 2).

At 02:00, he developed hypoxia, diaphoresis, confusion, and labored breathing with accessory muscle use concerning for airway compromise requiring rapid intubation. The intubation was without complication under etomidate and succinylcholine before maintaining sedation with propofol infusion. An initial arterial blood gas (ABG) was consistent with acute respiratory acidosis with a pH of 7.13, CO2 of 81.8, and bicarbonate of 20.0 mmol/L. The patient’s acidosis was initially improved on a repeat ABG; however, prior to his transfer to the ICU, he demonstrated significant dyssynchrony with the ventilator, and repeat ABG demonstrated worsening acidosis (Table 1).

**Table 1:** A visual depiction of serial arterial blood gas results during hospitalization. In the table, pH is measured as -log[H+], and time is depicted in hours and minutes. All samples were collected while the patient was on a pressure-controlled ventilation mode. FiO₂, fraction of inspired oxygen; HCO₃, bicarbonate; O₂, oxygen; pCO₂, partial pressure of carbon dioxide; pH, potential of hydrogen; pO₂, partial pressure of oxygen

pH	pCO₂ (mmHg)	pO₂ (mmHg)	O₂ saturation (%)	HCO₃ (mmol/L)	FiO₂ (%)	Time
7.13	81.8	394	97.0	20.0	100	03:48
7.24	61	208	100	26	100	04:55
7.11	77	401	100	24	100	08:52

**Figure 3:**
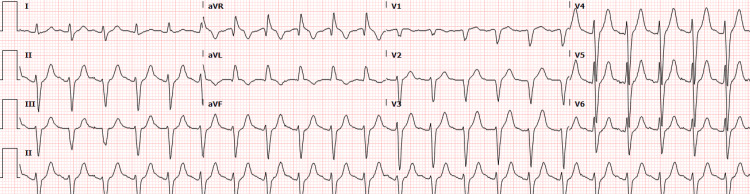
Repeat electrocardiogram demonstrating inferior ST-segment depressions during acute respiratory failure.

At this time, the patient was started on a ketamine drip for treatment of presumed bronchospasm and possible asthma exacerbation. A repeat EKG demonstrated ST-segment depression in the inferior leads, and troponin was elevated to 241 ng/L (Figure 3).

**Figure 4:**
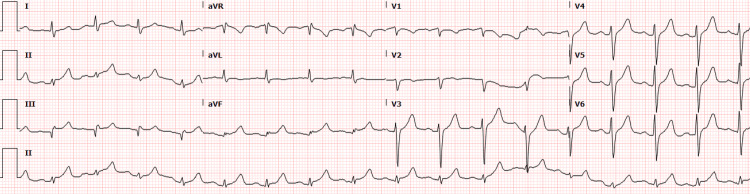
Repeat electrocardiogram, which then demonstrated new ST-segment depression in precordial leads.

**Figure 5:**
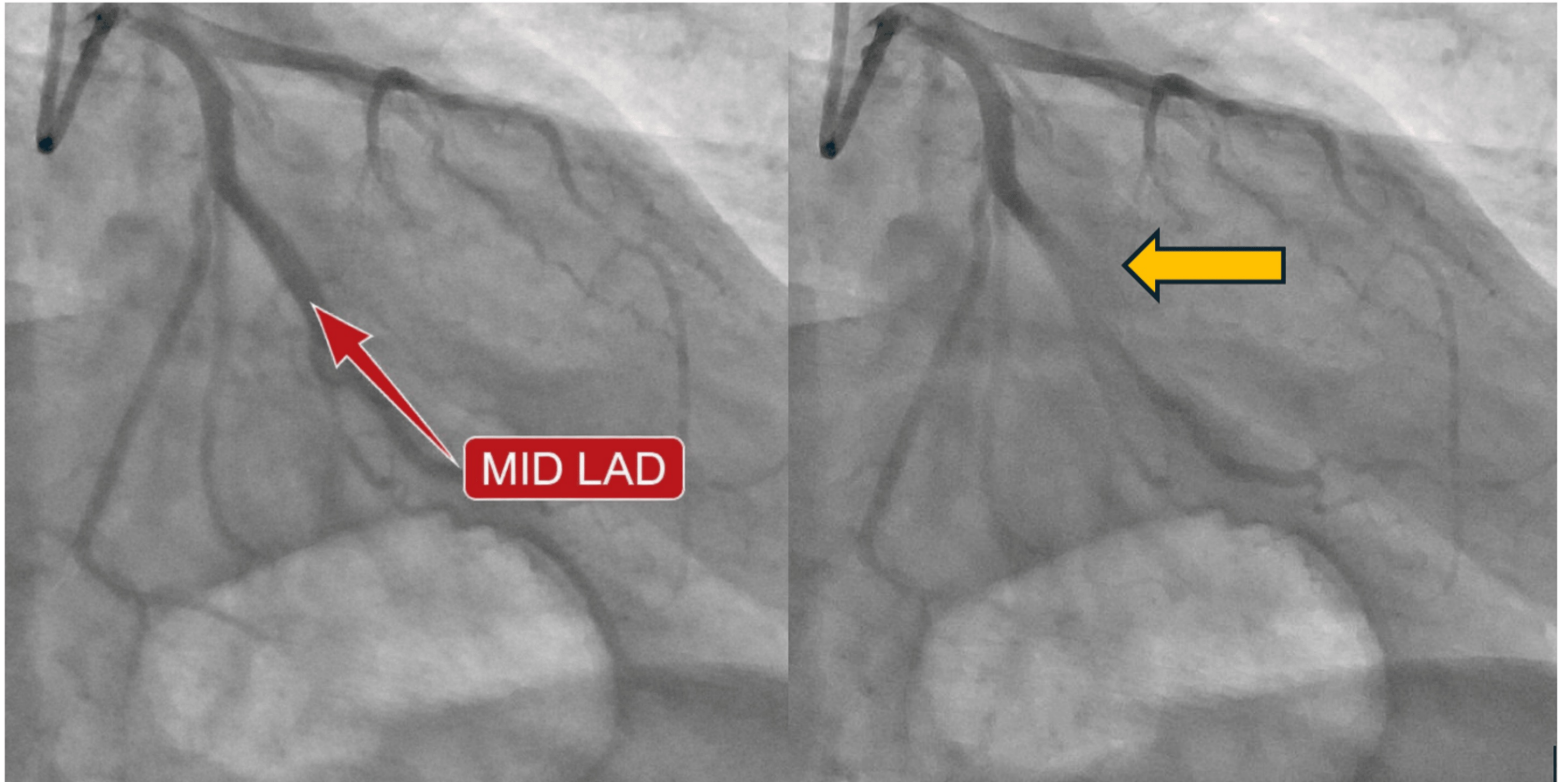
Images of the patient’s emergent cardiac catheterization demonstrating intermittent compression of the patient’s mid-left anterior descending artery (LAD). On the left, the patient’s LAD (red arrow) is seen to be patent during diastole, which is then seen to have decreased contrast filling during systole due to the segment within the myocardial bridge (orange arrow).

Repeat troponin showed further elevation to 281 ng/L and developing changes on an EKG to involve the precordial leads (Figure 4). He underwent emergent cardiac catheterization and was found to have a myocardial bridge in the mid-left anterior descending (LAD) artery (Figure 5). He continued to improve with the use of ketamine infusion and was extubated the following day. He was started on a prednisone taper, losartan 50 mg twice daily, and diltiazem 240 mg daily, and discharged with close outpatient follow-up.

## Discussion

Dyspnea is a common chief complaint; in the setting of a worsening wheeze, there is also the concern of possible new onset community-acquired pneumonia and undiagnosed chronic obstructive pulmonary disease (COPD). Initially, the mainstay of treatment is broad-spectrum antibiotics with a macrolide and cephalosporins. Acute bronchospasm leading to respiratory failure may result from infection, undiagnosed obstructive lung disease, environmental exposures, or iatrogenic triggers.

Immediate hypersensitivity reactions can be associated with cephalosporin use, typically presenting as urticaria, angioedema, and bronchospasm, and anaphylactic shock [6]. Crane et al. [6] presented a case where a patient developed hemodynamic instability after the administration of ceftriaxone, who developed recurrent bronchospasms. They reported that 1-4% of patients with a history of penicillin allergies develop cephalosporin sensitivity, which can develop into a life-threatening reaction [1,6]. The mechanism of these reactions is reported to be immunoglobulin E (IgE) mediated, while a definitive IgE-mediated reaction could not be established in the proposed case. However, the temporal association between ceftriaxone administration and respiratory decompensation alluded to medication-related bronchospasm as the trigger for clinical decline [1]. The sequence of events in this case would point to an immediate hypersensitivity reaction, given hypotension, tachycardia, wheeze, and dyspnea following the administration of ceftriaxone.

After the patient’s decompensation, he developed persistent ST-segment depression, troponin elevation, and subsequently underwent a cardiac catheterization, which revealed a myocardial bridge. Myocardial bridge is a typically benign congenital anomaly where a major coronary artery traverses myocardium and can be compressed during systole, leading to symptoms of acute coronary syndrome [4,5]. Occlusion can be partial or total, and the extent of arterial compromise, combined with increased heart rate or inotropic stimulation, may precipitate ischemia in predisposed individuals [4,7]. Choi et al. [8] presented a case of hemodynamic instability after the administration of general anesthesia secondary to coronary vasospasm and exacerbation of bridged section. In our case, we suspect the myocardial bridge contributed to troponin elevation, and during acute respiratory failure most consistent with a type 2 myocardial infarction due to inadequate oxygen delivery rather than plaque rupture [8].

Schwartz classification of myocardial bridge is based on symptom status: A is asymptomatic, B is symptomatic with signs of ischemia, and C is symptomatic with detection of vascular compromise on angiogram. Gómez-Moreno et al. presented a case of continued dyspnea and exertional chest pain unresponsive to pharmacological treatment, who was examined with a CT angiogram and found to have a myocardial bridge type B (Schwartz classification) [9]. This phenomenon is similar to the presented case; after the onset of bronchospasm, our patient developed symptoms with signs of ischemia, but these were not visible on the CT angiogram. It is typically diagnosed via coronary angiography, with a reported prevalence of 0.5-12.0% [4].

In the presence of bronchospasm or asthma, a diagnosis of a myocardial bridge imparts significant implications for diagnosis and treatment. With awareness of the myocardial bridge, the delineation of the airway from the cardiac etiology of chest discomfort can be made. Airway disorders should be considered in the presence of atopy or asthma, gradual onset, wheeze or hypoventilation, and improvement with bronchodilators. Cardiac etiology should be considered with a family history of cardiovascular disease, acute onset, palpitations, syncope, and no response to treatment.

In these instances, it is important to recognize myocardial bridge as a potential confounder in the setting of concurrent asthma, as it can lead to arrhythmia, ischemia, infarction, or death [4]. Nitrates and positive inotropic medications should be avoided due to augmentation of systolic compression of the bridge vessel. Treatment should target heart rate control, minimize the use of beta blockers during exacerbation, while considering the use of cardioselective medication to avoid bronchospasm. Lee et al. [10] postulated that vasodilation decreases coronary flow reserve, worsening damage, and thus allowing adequate filling time via cardioselective agents can reduce compression at the bridged area. These physiologic mechanisms, as well as albuterol-induced tachycardia, were likely apparent in our case [7,10].

## Conclusions

This case demonstrates the complex etiology of dyspnea due to ceftriaxone-induced bronchospasm resulting in first-time intubation and an exacerbation of an incidentally found myocardial bridge (Schwartz classification C). Highlighting that in instances of respiratory compromise, iatrogenic causes should be considered, and the offending agent should be stopped during patient stabilization. For patients with acute onset EKG changes, chest pain, or palpitations, while rare, a myocardial bridge should also be considered, and treatment should be tailored to avoid tachycardia and positive inotropic medications.
